# Molecular Evolution of Respiratory Syncytial Virus Fusion Gene, Canada, 2006–2010

**DOI:** 10.3201/eid1801.110515

**Published:** 2012-01

**Authors:** Jesse Papenburg, Julie Carbonneau, Marie-Ève Hamelin, Sandra Isabel, Xavier Bouhy, Najwa Ohoumanne, Pierre Déry, Bosco A. Paes, Jacques Corbeil, Michel G. Bergeron, Gaston De Serres, Guy Boivin

**Affiliations:** McGill University Health Centre, Montréal, Québec, Canada (J. Papenburg);; Centre de Recherche du Centre Hospitalier Universitaire de Québec, Québec City, Québec, Canada (J. Papenburg, J. Carbonneau, M.-È. Hamelin, S. Isabel, X. Bouhy, P. Déry, J. Corbeil, M.G. Bergeron, G. Boivin);; Institut National de Santé Publique du Québec, Québec (N. Ohoumanne, G. De Serres);; McMaster Children’s Hospital, Hamilton, Ontario, Canada (B.A. Paes)

**Keywords:** Human respiratory syncytial virus, children, fusion (F) protein, phylogeny, palivizumab, viruses, Canada

## Abstract

To assess molecular evolution of the respiratory syncytial virus (RSV) fusion gene, we analyzed RSV-positive specimens from 123 children in Canada who did or did not receive RSV immunoprophylaxis (palivizumab) during 2006–2010. Resistance-conferring mutations within the palivizumab binding site occurred in 8.7% of palivizumab recipients and none of the nonrecipients.

Human respiratory syncytial virus (RSV) is the most common cause of acute respiratory tract infections (RTIs) and a major cause of hospital admission and death among children <5 years of age worldwide (*1*). Risk for severe RSV-associated illness is highest among children born prematurely or with chronic medical disorders (*2*). Palivizumab immunoprophylaxis is the only available measure to prevent severe RSV disease.

The RSV fusion (RSV-F) surface glycoprotein mediates virus fusion to host cells. It is a major antigenic determinant that elicits neutralizing antibodies and cytotoxic T-lymphocyte immunity (*3*). Palivizumab (MedImmune, Gaithersburg, MD, USA) is a humanized mouse monoclonal antibody that inhibits RSV-F by binding to a defined epitope (residues 262–276) (*4**,**5*). Palivizumab immunoprophylaxis is recommended for the prevention of serious lower RTIs caused by RSV in children at high risk (*6*). RSV strains with mutations in key amino acid residues within the palivizumab binding site are resistant to this antibody (*7**–**9*); however, little is known about the prevalence of such mutations in clinical samples. Furthermore, despite its role in RSV pathogenesis, immunity, and prevention strategies, few data on RSV-F molecular evolution are available (*10**,**11*) because previous phylogenetic studies have focused on the RSV-G glycoprotein (*12**,**13*). Therefore, we monitored evolutionary changes in RSV-F, particularly potential resistance mutations in the palivizumab binding site, among strains from children who did and did not receive palivizumab.

## The Study

This cohort study was approved by the Centre Hospitalier Universitaire de Québec Research Ethics Board. Participants were <3 years of age and either received medical attention at an outpatient pediatric clinic or were hospitalized at a pediatric center for acute RTI during 4 winter seasons (2006–2010), in Québec City, Québec, Canada.

Clinical data were prospectively collected at study entry and after 1-month follow-up. For all patients, at the first visit a nasopharyngeal aspirate was collected. The aspirate was frozen at −80°C until subsequent testing by a multiplex PCR/DNA hybridization assay that detects RSV genotype-A (RSV-A), RSV-B, and 22 other respiratory viruses (Infiniti RVP assay; Autogenomics, Carlsbad, CA, USA) (*14*).

RSV infection was identified in aspirates from 467 (63.6%) of 734 hospitalized children (257 RSV-A, 210 RSV-B) and from 147 (48.2%) of 305 outpatient children (85 RSV-A, 62 RSV-B). During 2006–2010, a total of 724 children received palivizumab in the Québec City region (L. Cliche, pers. comm.). RSV-positive samples from all 12 study participants receiving palivizumab and from 100 not receiving palivizumab underwent RSV-F sequencing.

Additionally, F-gene analysis was performed on 11 RSV-positive clinical samples from palivizumab recipients retrospectively identified by using neonatal clinic registries at McMaster Children’s Hospital (Hamilton, Ontario, Canada) and Montréal Children’s Hospital (Montréal, Québec, Canada) during 2009–2010. Clinical data were collected by chart review.

RNA was extracted directly from nasopharyngeal samples by using a QIAmp Viral RNA Mini Kit (QIAGEN, Mississauga, Ontario, Canada). Random primers (Amersham, Piscataway, NJ, USA) and Superscript II RT Kit (Invitrogen, Carlsbad, CA, USA) were used for reverse transcription. PCR amplification was performed with QuantiFast Probe PCR+ROX Vial Kit (QIAGEN); primers and thermocycling conditions are available from G.B. upon request. RSV-F amplicons were sequenced by using an automated sequencer (Applied Biosystems, Foster City, CA, USA).

Along with the newly generated nucleotide sequences from the 123 children (23 palivizumab recipients; 100 nonrecipients), we analyzed 92 clinical RSV-F sequences provided by other investigators (*10**,**11*) or available from GenBank. Palivizumab exposure was unknown for these samples, and all originated outside Canada. We also included 10 unpublished RSV-F sequences from a 2004–2005 study of palivizumab recipients in Canada (*15*). Multiple-sequence alignment was performed with ClustalW in MEGA5 (www.megasoftware.net). A 1,524-bp region (positions 79–1602 in the prototype A2 and B1 RSV-F genes) was translated into amino acid sequences, and the palivizumab binding site (residues 262–276) was assessed for variations. We removed 55 redundant (identical) nucleotide sequences. The final dataset comprised 170 unique sequences, including 89 that were newly generated in this study (GenBank accession nos. JF776691–JF776779).

Phylogenetic reconstructions were computed in MEGA5 (Figure). Strains from Canada tended to group together. However, this segregation likely reflects temporal evolution rather than geographic influence because all RSV-F sequences originating from other countries were collected >10 years ago. Previous RSV-G studies have demonstrated concurrent circulation of related lineages in distant areas (*13*). Overall, RSV-F was highly conserved (Table 1). RSV-A exhibited more genetic diversity than RSV-B. This difference may reflect sampling bias because more RSV-A sequences originating from diverse locations and years were available; however, the greater genetic diversity of RSV-A compared with RSV-B has also been observed in a study conducted in South Korea (*10*).

**Figure:**
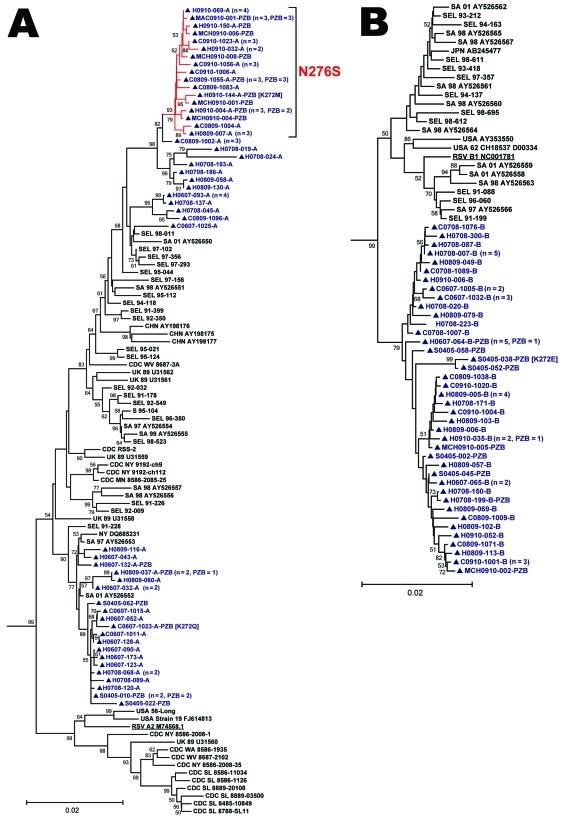
Phylogenetic analysis of 170 near–full-length unique respiratory syncitial virus fusion (RSV-F) gene sequences (nt 79–1602). Panels A and B are detailed phylograms of the RSV-A and RSV-B taxa analyzed, respectively. One bovine RSV-F sequence was added to the dataset (GenBank accession no. AF295543.1) as the outgroup (not shown) and used for rooting the phylograms. Topology was inferred by using the neighbor-joining method, and evolutionary distances were computed by using the maximum-composite likelihood method in MEGA5 software (www.megasoftware.net). The topologic accuracy of the tree was evaluated by using 1,000 bootstrap replicates. Only bootstrap values >50% are shown. Blue text and triangles represent RSV strains isolated in Canada and sequenced at the Centre de Recherche du Centre Hospitalier Universitaire de Québec; red branches indicates a sublineage of RSV-A with a N276S mutation; underlining indicates prototypical RSV A2 and RSV B1 strains. The clinical origin of strains from Canada (prospective study hospitalized patient [H] or clinic patient [C]; 2004–2005 palivizumab study patient [S]; retrospectively identified patient from the Montréal Children’s Hospital [MCH] or McMaster Children’s Hospital [MAC]) is indicated, followed by the year collected, the specimen identifier, and the result of RSV genogroup testing (RSV-A [A], RSV-B [B]) by multiplex PCR/DNA hybridization assay (*14*). Specimens with a nonsilent mutation at codon 272 have the amino acid substitution identified in brackets. When a taxon represents >1 identical sequences, the number of patients that it represents and the number of palivizumab recipients (PZB) among these patients are noted in parentheses. SA, South Africa; CHN, People’s Republic of China; UK, United Kingdom, JPN, Japan; AUS, Australia; USA, United States; CDC, US Centers for Disease Control and Prevention; NY, New York; SL, St. Louis; WV, West Virginia; MN, Minnesota; MD, Maryland; WA, Washington; SEL, Seoul, South Korea. Scale bars represent substitutions per basepair per the indicated horizontal distance.

**Table 1:** Estimated mean nucleotide and amino acid identities of 170 unique respiratory syncytial virus fusion gene sequences*

Sequence	Mean nucleotide identity, % ± SD	Mean amino acid identity, % ± SD
Overall, n = 170	89.59 ± 8.38	95.99 ± 3.25
RSV-A (within group), n = 105	97.06 ± 1.33	98.85 ± 0.76
RSV-B (within group), n = 65	98.65 ± 0.69	99.48 ± 0.28
RSV-A vs. RSV-B (between groups), n = 170	80.85 ± 0.66	92.64 ± 0.56

*Mean nucleotide and amino acid identities were estimated by calculating pairwise distances in MEGA5 software (www.megasoftware.net) and using the maximum-composite likelihood and Poisson correction models, respectively. RSV, respiratory syncytial virus.

Of the 23 RSV-F amino acid sequences from patients who received palivizumab (Table 2), 2 exhibited a mutation at residue 272 (K272Q, K272M), whereas no such mutations were identified in the other 100 strains obtained from those who did not receive palivizumab (8.7% versus 0%; p = 0.03, by Fisher exact test). A sublineage of RSV-A, noteworthy for an N276S substitution in the palivizumab binding site, emerged during 2008–2009 (8 [44%] of 18 RSV-A strains) and became the predominant RSV-A clade during 2009–2010 (25 [100%] of 25 strains) in palivizumab recipients and nonrecipients. This N276S lineage was detected in all 3 Canadian communities studied. No sequences from GenBank or other studies (*10**,**11*) harbored any palivizumab binding site mutation.

**Table 2:** Characteristics of 23 children in whom clinically significant respiratory syncytial virus respiratory tract infection developed while receiving palivizumab immunoprophylaxis, Canada, 2006–2010*

Location and patient ID	Age, mo/ sex†	GA at birth, wk + d	Underlying comorbidities	No. doses PZB‡	Delay, d§	Clinical diagnoses	H	Multiplex PCR/DNA results	Mutation
Québec City, Québec (2006–2010)									
C0607-1023	9/F	32 + 4	Prematurity, LBW	3	21	Bronchiolitis	No	RSV-A; enterovirus type A	K272Q
H0607-064	24/M	38 + 3	Congenital myopathy	3	15	Pneumonia; bronchospasm	Yes	RSV-B	NF
H0607-132	12/M	38 + 5	Pulmonary artery stenosis	5	7	Bronchiolitis	Yes	RSV-A	NF
H0708-199	4/M	30 + 4	Prematurity, VLBW	4	14	Bronchiolitis	Yes	RSV-B	NF
H0809-037	11/F	27 + 5	Prematurity, ELBW	3	14	Bronchiolitis	Yes	RSV-A	NF
C0809-1055	6/F	29 + 0	Prematurity, ELBW, triplet	4	27	Bronchiolitis	No	RSV-A	N276S
C0809-1056	6/M	29 + 0	Prematurity, ELBW, triplet	4	27	Bronchiolitis	No	RSV-A	N276S
C0809-1057	6/M	29 + 0	Prematurity, VLBW, triplet	4	27	Bronchiolitis	No	RSV-A	N276S
H0910-004	4/F	39 + 5	Choanal hypoplasia	1	16	Apnea; upper RTI	Yes	RSV-A	N276S
H0910-140	6/M	25 + 5	Prematurity, ELBW	4	29	Bronchiolitis	Yes	RSV-B	NF
H0910-144	13/F	26 + 5	Prematurity, VLBW	4	7	Pneumonia	Yes	RSV-A	K272M, N276S
H0910-150	9/M	28 + 3	Prematurity, VLBW	4	12	Upper RTI; acute otitis media	Yes	RSV-A; adenovirus type C	N276S
Montréal, Québec (2009–2010)									
MCH0910-001	15/M	40 + 4	Total anomalous pulmonary venous return	3	26	Pneumonia	Yes	RSV¶	N276S
MCH0910-002	6/F	39 + 0	Pulmonary valve stenosis, right aortic arch	2	7	Bronchiolitis	Yes	RSV¶	N276S
MCH0910-003	5/M	39 + 6	Cystic fibrosis	3	24	Bronchiolitis	No	RSV¶	N276S
MCH0910-004	7/M	36 + 2	Prematurity, BPD hypotonia	4	6	Bronchiolitis	Yes	RSV¶	N276S
MCH0910-005	15/M	40 + 4	Neuromuscular disorder, recurrent aspirations	4	13	Upper RTI; acute otitis media	No	RSV¶	N276S
MCH0910-006	2/M	34 + 6	Prematurity, LBW	1	14	Bronchiolitis	Yes	RSV¶	N276S
MCH0910-007	19/F	25 + 0	Prematurity, ELBW, BPD	3	19	Bronchiolitis, bronchospasm	Yes	RSV¶	N276S
MCH0910-008	2/F	38 + 1	Neuromuscular disorder, ventricular septal defect	2	12	Bronchiolitis	Yes	RSV¶	N276S
Hamilton, Ontario (2009–2010)									
MAC0910-001	1/F	34 + 5	Prematurity, LBW	2	3	Bronchiolitis	Yes	RSV¶	N276S
MAC0910-002	6/F	34 + 3	Prematurity, LBW, twin	1	25	Bronchiolitis	Yes	RSV¶	N276S
MAC0910-003	6/F	34 + 3	Prematurity, VLBW, IUGR, twin	1	27	Bronchiolitis	Yes	RSV¶	N276S

*Patient identification (ID) nomenclature: hospitalized (H) or clinic (C) prospective study participant, Montréal Children’s Hospital (MCH) or McMaster Children’s Hospital (MAC) patient. GA, gestational age; PZB, palivizumab; multiplex PCR/DNA, hybridization assay; mutation, mutation in respiratory syncytial virus fusion protein PZB binding site (residues 262–276); RSV, respiratory syncytial virus; LBW, low birthweight (1,500–2,500 g); NF, no mutation found in PZB binding site; VLBW, very low birthweight (1,000–1,499 g); ELBW, extremely low birthweight (<1,000 g); RTI, respiratory tract infection; BPD, bronchopulmonary dysplasia; IUGR, intrauterine growth restriction. †Median patient age 6.0 mo (range 1–24 mo). ‡Mean ± SD no. palivizumab doses received that winter: 3.0 ± 1.2 doses. §Median interval between last palivizumab dose and symptom onset: 15.0 d (range 3–27 d). ¶Retrospectively identified participants from Montréal Children’s Hospital or McMaster Children’s Hospital were RSV-positive by direct immunofluorescence assay (Chemicon International, Temecula, CA, USA) and were not tested by the multiplex PCR/DNA hybridization assay (*14*).

Microneutralization assays were performed as described elsewhere, with minor modifications (*9*). RSV was incubated (for 2 h at 37°C) with serially diluted palivizumab, then cultured in Vero cells (for 5 d). RSV replication and 50% inhibitory concentrations (IC_50_) were subsequently determined by F protein quantification by using ELISA. The mean ± SD IC_50_ of C0910–1006A, a N276S strain, was 0.33 ± 0.04 µg/mL, similar to that of RSV-A2 wild type (IC_50_ 0.46 ± 0.04 µg/mL) and therefore was considered susceptible. Position-272 variants did not grow in culture and were not tested.

## Conclusions

We report the prevalence of resistance-conferring mutations in RSV-F among children receiving or not receiving palivizumab. Although infrequent (8.7% of infections in palivizumab recipients), residue 272 mutations were significantly associated with palivizumab exposure and not observed at all in nonexposed patients.

We identified 2 new clinical specimens with position 272 mutations (K272Q and K272M). We cannot exclude the possibility that additional specimens contained mixed viral populations with minor proportions of position-272 mutants not detectable by conventional sequencing methods. Changes at this position (from lysine to asparagine, glutamine, glutamic acid, methionine, or threonine) have produced palivizumab resistance in vitro (*9*) and in a cotton rat model (*7*). As previously reported, 1 (10.0%) of 10 sequences from our 2004–2005 study of palivizumab patients carried a 272 mutation (K272E) (*15*). The K272E substitution is the only substitution also demonstrated to confer resistance to motavizumab, an enhanced-potency monoclonal antibody developed by affinity maturation of palivizumab (*9*). We could not perform neutralization assays on position-272 variants because they did not grow in culture. This finding suggests that such changes adversely affect viral replicative capacity (*7**,**9*).

Phylogenetic analysis demonstrated that mutations in the palivizumab binding site occur in diverse genetic backgrounds; all 3 strains with substitutions at residue 272 grouped to different clades (Figure). Furthermore, these mutant strains caused mild disease treatable in an outpatient clinic and severe illness requiring hospitalization.

From 3 Canadian communities we detected a lineage harboring an N276S mutation in 44.4% of RSV-A sequences from 2008–2009 and 100% from 2009–2010, unrelated to palivizumab exposure. Adams et al. have proposed that N276S led to palivizumab resistance in a clinical specimen (*8*). However, that sample also comprised a K272E subpopulation. Our microneutralization assay results and unpublished neutralization data using recombinant viruses and clinical isolates (Q. Zhu, pers. comm.) suggest that N276S does not confer resistance.

Although serious RSV RTIs during palivizumab prophylaxis remain uncommon, we observed an 8.7% prevalence of known resistance mutations among 23 medically attended patients receiving palivizumab. These findings underscore the need for continued monitoring of RSV-F evolution.
